# Ocular surface symptoms among individuals exposed to ambient levels of traffic derived air pollution – a cross-sectional study

**DOI:** 10.12688/f1000research.13483.2

**Published:** 2018-09-18

**Authors:** Nabin Paudel, Sanjeev Adhikari, Sarina Manandhar, Ashesh Acharya, Ajit Thakur, Bhairaja Shrestha

**Affiliations:** 1 Drishti Eye Care Center, Kathmandu, 44620, Nepal

**Keywords:** Air pollution, ocular surface, OSDI questionnaire, Kathmandu, Dry Eye

## Abstract

**Background: **The ocular surface is separated by a thin layer of tear film from outdoor air pollutants making individuals exposed to outdoor air pollution prone to various ocular surface disorders. The aim of this study was to determine the magnitude of ocular surface disorders symptoms among traffic police officers of Kathmandu, Nepal.

**Methods:** Two hundred traffic police officers working at different traffic police office branches of Kathmandu, Nepal were invited to the police headquarters for eye and vision examination. Among them, 91 individuals (95% males) completed the ocular surface disease index (OSDI) questionnaire and underwent Schirmer’s I tear test.

**Results:** Symptoms of ocular surface disorders were reported by over 80% of the individuals. Approximately two-fifths of the individuals (38%) reported severe symptoms.  Only 17% of the individuals’ tear secretion was found to be below normal using the Schirmer’s tear test. No significant association was observed between the OSDI score and Schirmer’s tear test scores (r = 0.008, p = 0.94). A weak but significant relationship was observed between the OSDI score and job duration (r=0.21,p = 0.04). Individual exposed to outdoor air pollution for more than 10 years had higher odds of reporting ocular surface complaints as compared to those who were exposed for less than 10 years (OR = 3.94, p = 0.02).

**Conclusion:** Ocular surface disorder symptoms are common among traffic police officers of Kathmandu, Nepal. The duration of exposure appears to significantly contribute to the increased symptoms in this exposed population.

## Introduction

Studies conducted so far on air pollution and the human ocular surface have demonstrated a link between air pollution and ocular discomfort, abnormal tear structure, and ocular surface inflammation ^1^. There are only a handful of studies demonstrating the association between the signs and symptoms of the ocular surface with air pollution ^2, 3^. Studies are even more infrequent from cities in developing countries, where the concentration of air pollutants in the environment is on rise. Air pollutants include minute particles such as particulate matter (PM), ozone (O _3_), nitrogen dioxide (NO _2_) and sulphur dioxide (SO _2_). The most common measures of air pollution are the concentration of PM _2.5 _and PM _10_ in the air. PM _2.5 _denote particulate matter that is smaller than 2.5 um and PM _10 _denote particulate matter less than 10 um. Sources of air pollution include combustion of wood and fossil fuels, road transport, forest fires, road and building construction ^4^. In Kathmandu, road transport is the main cause of air pollution. Over the last 20 years there has been a rapid increase in the number of vehicles in the Kathmandu valley. The increasing purchase of private vehicles due to inefficient public transport system may have led to the increase in the number of vehicles. From a cumulative number of 20,000 vehicles registered in the year 2000, the latest number of vehicles registered in the Kathmandu Valley has reached over 90,000 in the year 2015/16 ^5^. Kathmandu is considered as one of the most highly polluted cities in the world, and Nepal is listed as one of the most polluted countries according to the WHO urban air pollution database. The annual average of the PM _2.5_, CO and NO _2_ concentration of Kathmandu for the year 2015 was 49 µg/m ^3^ (range, 24–70), 438 µg/m ^3^ (range, 298 – 517) and 176 µg/m ^3^ (range, 47 – 315) with a maximum concentration during the winter season ^6^. Similarly, even though the annual average value of black carbon (BC) is not available, a study conducted in Kathmandu in 2014 reported the levels of BC between 16.74 µgC/m ^3^ – 16.74 µgC/m ^3^ during the duration of their data collection period ((16 February–4 April 2014) and (20 July–22 August 2014)) ^7^. These values are clearly higher than the recommended level which raises a significant concern regarding the health of people exposed to these conditions.

Traffic police officers in Kathmandu are prone to air pollution related disorders as they spend most of their time outdoors at various intersections controlling the flow of vehicles as modern electronic traffic management systems are unavailable in the city. Plenty of previous studies have reported a higher prevalence of respiratory disorders and early biological changes in the DNA ^8^ of traffic officers who are exposed to air pollution as compared to control population ^9^. However, very little attention has been paid on the ocular effect of air pollution in this population. Therefore, the purpose of this study was to determine the magnitude of ocular surface disorders based on a subjective symptoms questionnaire and a commonly used tear secretion test (Schirmer’s I test) in traffic police officers of Kathmandu, Nepal. In addition, the association between the two tests in this population was explored.

## Methods

### Study population

This study involved a cross-sectional, community-based assessment on 91 traffic police officers (86 male, 5 female) recruited among the officers of the Traffic Metropolitan head office, Baggikhana, Kathmandu, Nepal. The participants were invited by word of mouth by the head officer along with a formal written notice. Participants with any chronic illness, smoking habit, taking any systemic drugs, having any ocular diseases, previous ocular surgery and current contact lens wear were excluded from the study. All of the individuals had presenting visual acuity of better than 20/25 at both near and far. Only those participants who met our inclusion criteria and agreed to participate were included in the study. The study was conducted in the month of August 2017.

### Ethics and consent

The study protocol was approved by the Ethics Committee of the Nepal Health Research Council (Reg.No.218/2017). The study was part of a larger program that was aimed at determining ocular and visual disorders in police officers. All of the research participants provided their written informed consent for participation before being enrolled in the study. The Declaration of Helsinki was followed while assessing the participants.

### Ocular Surface Disease Index (OSDI) Questionnaire

The Ocular Surface Disease Index Questionnaire ^10^ is a validated tool to assess the subjective symptoms of individuals with potential ocular surface disorders. It consists of a total of 12 items that asks questions to individuals about their symptoms and their exposure to environmental risk factors in the last week. ( Supplementary File 1) The Nepali translated OSDI questionnaire was administered to all of the participants before conducting the clinical assessment. The OSDI score was calculated using the following formula:

(Sum of scores for all questions answered) X 25

      (Total number of questions answered)

An OSDI score of 0–12 was considered normal, 13–22 as mild, 23–32 as moderate, and 33–100 as a severe ocular surface disorder ^11^.

### Schirmer I test

The Schirmer I test, a commonly used tear secretion test utilises a special filter paper (Whatman filer paper) (5mm X 35mm) that is placed in the eye with the eye open or closed. The amount of moisture (measured as the length of the paper that is wet) that the paper collects over a 5 minute duration determines the severity of dry eye.

The Schirmer I tear test was conducted under topical anaesthesia (0.5% Proparacaine). The test was conducted in an indoor setting at room temperature. After instilling one drop of proparacaine in each eye, the eye was dried with cotton for any residual drop. The Schirmer strip was then placed on the lateral 1/3rd aspect of the lower eye lid taking special care not to touch the cornea. The strip was removed from the lid after 5 minutes. The Schirmer’s test value is variable in normal individuals making it hard to determine cut-off value between normal and abnormal. The measurement of 5mm or less was considered as abnormal based on its better diagnostic accuracy in determining dry eye patients ^12^. 

### Ophthalmological examination

Routine ophthalmological examination including visual acuity, refraction, anterior segment and posterior segment assessment was conducted. These variables were, however, not analysed as a part of this study

### Other variables

Other variables such as age, gender, and duration of working as a traffic officer were also recorded.

### Statistical analysis

Data are presented as mean±SD unless mentioned otherwise. Independent sample t-test was employed to compare the mean between two groups whereas one way ANOVA, along with appropriate posthoc tests, was employed to compare the means between three or more groups. Paired t-test was employed to compare test results between the two eyes. Pearson correlation was employed to determine the association between variables. Binary logistic regression analysis was also employed to determine association between dependent and independent variables. Statistical analysis was conducted using SPSS V22, IBM, California.

## Results

**Figure 1.**
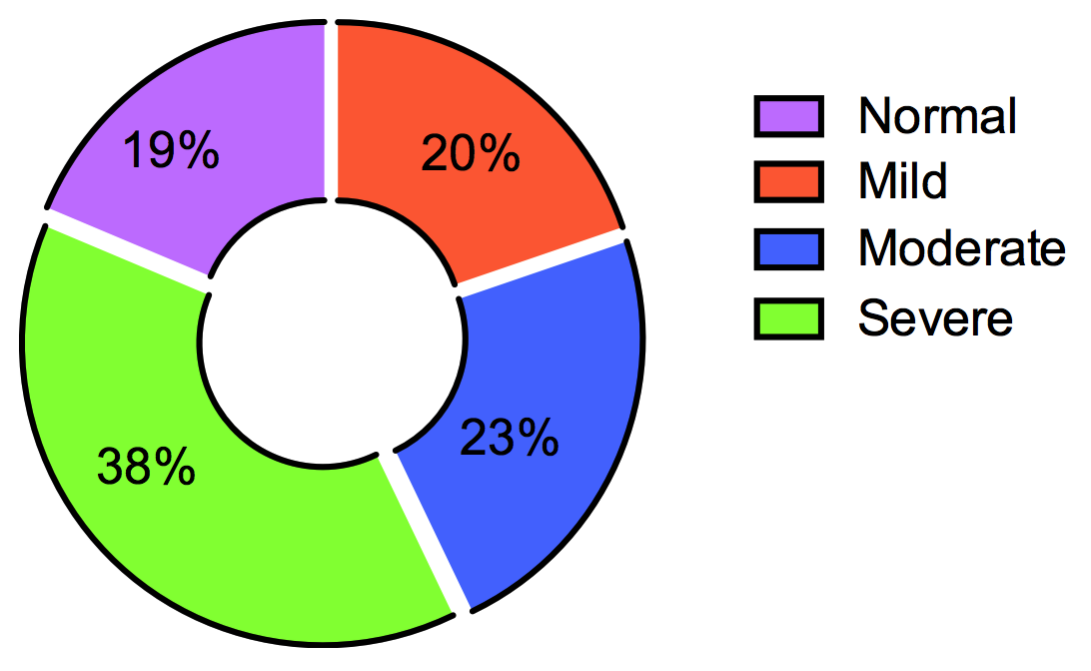
Frequency of ocular surface disorder symptoms according to the OSDI score.

The characteristics of participants used in this study are shown in Table 1. The mean age of the participants was 32±6 years. The OSDI questionnaire was completed by all subjects. The mean OSDI score was 30.11±19.70 (range 2 to 97.90). Based on the OSDI score, 81% of the participants reported symptoms of ocular surface disorder; over one third (38%) of the participants reported symptoms of severe ocular surface disorder ( Figure 1).

**Table 1.** Characteristics of the patients enrolled in the study.

Characteristics (values are expressed as mean± SD unless mentioned otherwise)
**Age** **32 ± 6 years** **Age Group** **<30 years – 36 (39.5%)** 30–40 years – 45 (49.5%) **>40 years – 10 (11%)** **Gender** **Male – 86 (94%)** **Female – 5 (6%)** **OSDI Score** **30.12 ± 19.70** **Schirmer’s RE** **16.12 ± 10.4 2 mm** **Schirmer’s LE** **17.43 ± 10.84 mm** **Duration of holding the job** **11.10 ± 5.8 years** **Category - job duration** Less than 5 years – 13 (14%) 5–10 years – 27 (30%) >10 years – 51 (56%) **Visual Acuity RE** **0.05 ±0.09 logMAR** **Visual Acuity LE:** **0.06 ± 0.11 logMAR**

Schirmer’s test of both eyes was conducted in all of the enrolled participants. The mean ± SD Schirmer’s test value (mm) for right eye (RE) and left eye (LE) was 16.12±10.42 and 17.42±10.84, respectively. There was a high correlation (r= 0.80, p<0.001) but a non-significant difference (p=0.08) in the Schirmer's test score between the two eyes. Hence the results of the right eye and left eye were averaged for analysis. Only 14% of the subjects’ Schirmer’s score showed abnormal results.

**Figure 2.**
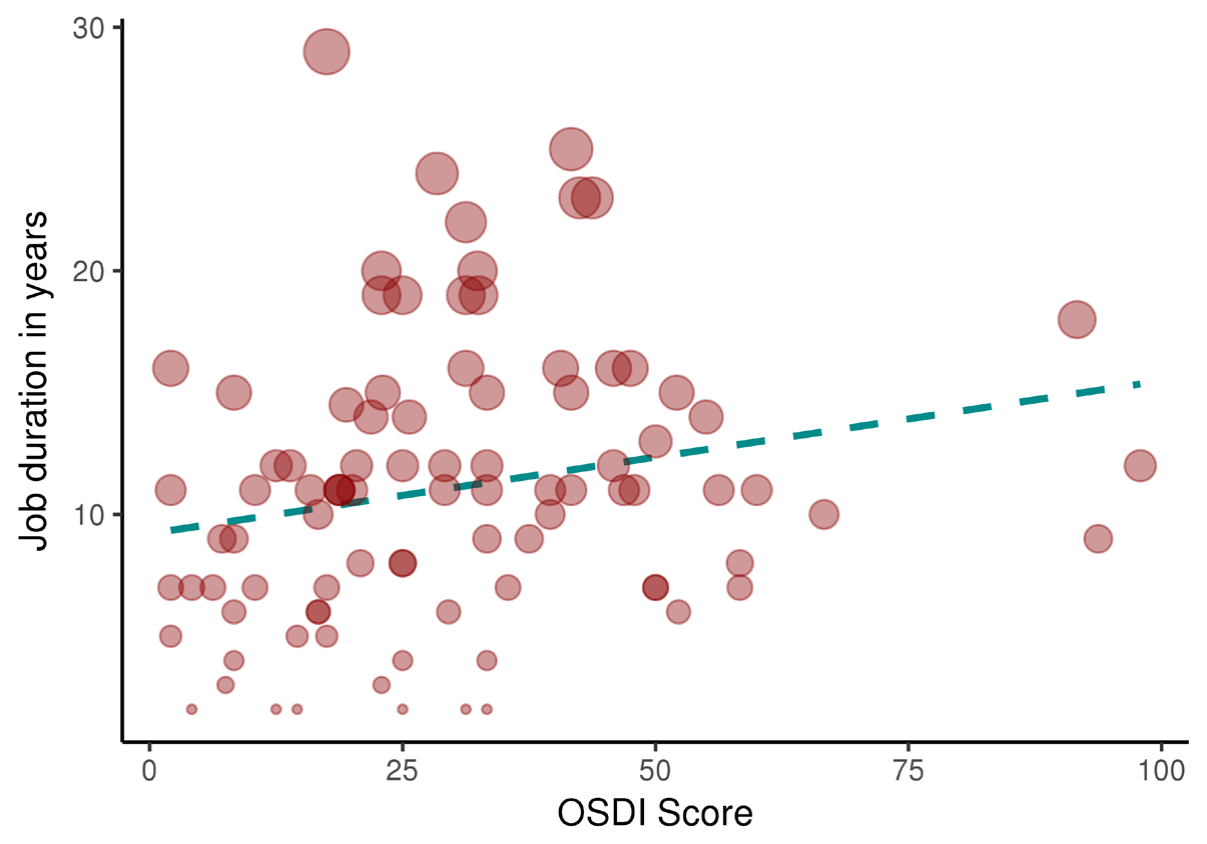
Correlation between OSDI and job duration. The circles in the graph are proportional to job duration (larger the circles longer is the job duration).

No association was observed between the OSDI score and the Schirmer’s test results (r=0.008, p=0.94). No significant correlation was also observed between the OSDI scores and age (r=0.15, p=0.14). A weak, but statistically significant, positive correlation was observed between OSDI score and duration of work (r=0.21, p=0.04) ( Figure 2)

**Figure 3.**
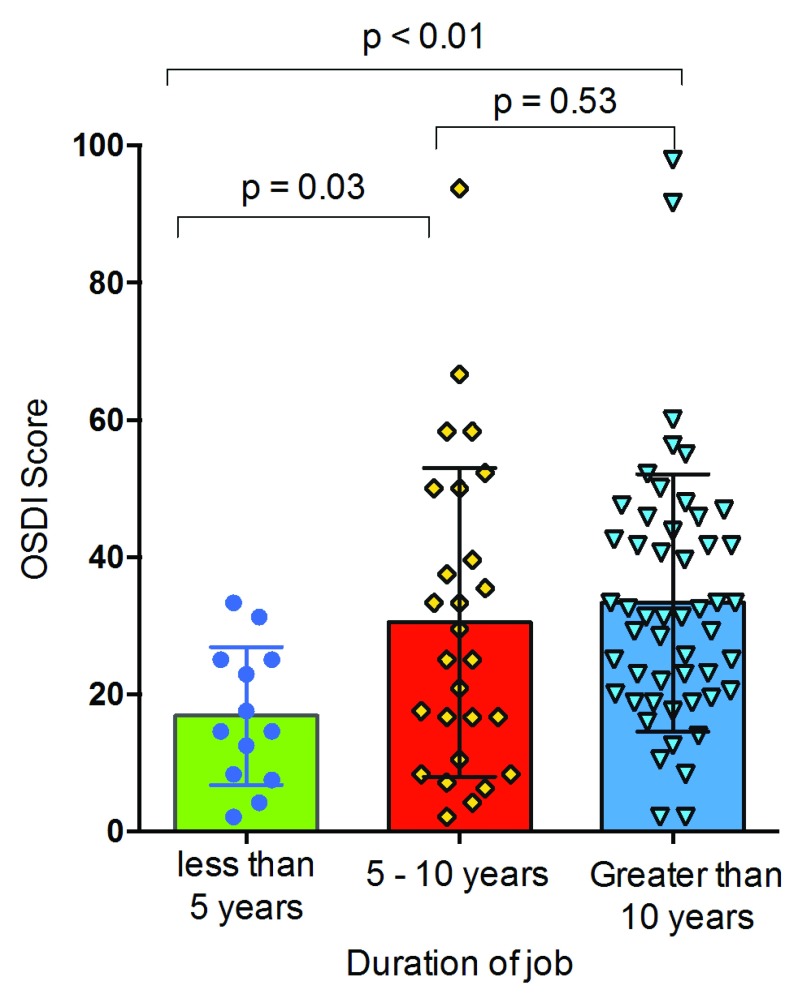
Variation of the OSDI score according to the duration of the job. (Bars represent mean score and error bars represent standard deviation.)

The mean duration of work was 11±6 years. Individuals who had held the job for more than 5 years had severe symptoms, as compared to those who had held the job for less than five years (p=0.001). A one way ANOVA test demonstrated a significant difference in the OSDI score between different age groups (<30, 30–40 and >40 years) (F _2,88 _= 3.86, p=0.025). The symptoms score was statistically significantly different between individuals who had worked for up to 5 years, five to ten years (mean difference, 13.65 ± 6.44, 95% CI, 0.85 to 26.46) and more than 10 years (mean difference, 16.48± 5.93, 95% CI, 4.70 to 28.27). However, no statistically significant difference was observed between individuals who had held the job for 5–10 years and >10 years (mean difference 2.82 ± 4.54, 95% CI, - 6.20 to 11.50) ( Figure 3). Furthermore, individuals who held the job for 10 years or more had significantly higher odds of having ocular surface symptoms as compared to those who had the job for less than ten years (OR: 3.94, 95% CI, 1.25-12.8, p = 0.02). There was a slight increase in the odds of having ocular surface symptoms after adjusting for age and gender, but it was borderline significant (OR: 4.28, 95% CI, 0.93-19.58, p = 0.05)

Of the 74 subjects identified as having symptoms of ocular surface disorders according to the OSDI score, only 16 were identified as abnormal by the Schirmer’s test.

Data on the ocular surface symptoms among individuals exposed to ambient levels of air pollution Click here for additional data file. Copyright: © 2018 Paudel N et al. 2018 Data associated with the article are available under the terms of the Creative Commons Zero "No rights reserved" data waiver (CC0 1.0 Public domain dedication).

## Discussion

This study explored the symptoms of ocular surface disorders among individuals exposed to traffic-derived air pollution in Kathmandu, Nepal. A remarkable proportion of individuals reported symptoms with over one third reporting symptoms of severe ocular surface disorder. Ocular surface disorder vary with age, whereby the prevalence is 11% among individuals between 40 to 59, and 18% in individuals above 80 ^13^. In this study, individuals were between 18 to 48 years, and 80% had symptoms of OSD, which is alarmingly high as compared to the general population ^13^.

Previous reports exploring symptoms in individuals exposed to traffic-derived air pollution have found mixed results. The Torricelli *et al*. ^14^ study in a group of 71 taxi drivers and traffic controllers reported that most of their subjects reported few symptoms, and fell within the normal category according to the OSDI scoring. However, they demonstrated that objective tests such as tear osmolarity and break up time were significantly reduced. In contrast, Saxena *et al*. reported that most of the subjects who were exposed to air pollution had more symptoms (irritation, itching, lacrimation, and redness) as compared those who were not exposed ^2^.

A majority of the individuals’ Schirmer’s results were within normal range in the present study. Similar normal findings of Schirmer’s test have been found by previous researchers ^12, 14^. This finding is not surprising as the poor diagnostic ability of the Schirmer’s test for detecting ocular surface dysfunction has been well recorded in the literature ^15^. The Schirmer’s test has shown normal results in many previous studies conducted among established dry eye population ^15^.

The lack of correlation between the OSDI scores and the Schirmer’s results is also not surprising, as this finding is consistent with most of the previous studies where the signs and symptoms of ocular surface disorders, particularly that of the dry eyes, are not correlated with one another ^16^. It is postulated that dry eye is a multifactorial disorder, and different mechanisms and factors act in compliment or may act independently to elicit the symptomatology of this condition ^17^.

The weak but statistically significant positive correlation between the OSDI score and duration of holding the current job (years) suggests that the longer the exposure, the more severe the symptoms. However, the finding that the mean symptom score is not significantly different between individuals who have held the job for 5–10 years and in those over 10 years signifies that exposure to ambient air pollution over 5 years poses a significant impact on the ocular surface. Furthermore, the higher odds of having ocular symptoms in individuals with over 10 years of holding the job suggests that the effect of air pollution on the ocular surface may have a cumulative effect over the years until symptoms start to appear.

Nepal was ranked as the 177th country just above China, Bangladesh, and India among the 180 countries with air quality issues according to the Environmental Performance Index (EPI) of 2016 ^18^. A report in 2007 on the air pollution concentration, specifically of the PM _2.5_ of the Kathmandu valley, was found to be 17-18 fold higher than the recommended 25ug/m ^3^ threshold provided by the WHO. A 2016 air pollution report of Nepal provided by the WHO has shown a considerable increase in PM _2.5 _concentration over a decade ^19^. Our study was conducted in the month of August 2017. The mean 24-hour average PM _2.5 _concentration during that month was 113.5 ug/m ^3^(approximately 5 fold higher than that recommended by the WHO) and the PM _10_ concentration was 633ug/m ^3^(approximately 13 fold higher than the WHO recommendation) (see Kathmandu Air Pollution: Real-time Air Quality Index and Department of Environment, Air Quality Monitoring). In light of the high levels of air pollution of Kathmandu, the higher number of individuals reporting severe symptoms of ocular surface disease in our study can be explained. Furthermore, the month of August is generally hot (average temperature, 29 degrees) and humid (average humidity, 83%) in Kathmandu. People often use fan and air conditioning indoors. In addition, there is in increase in the allergens in the environment during this season that may lead to an increased frequency of itching, foreign body sensation and photophobia. All these factors may also have contributed to the increasing symptomatology of officers enrolled in this study.

While this study provided novel ocular health issues in this more exposed population, some limitations must be acknowledged. Firstly, only two tests – the OSDI questionnaire and the Schirmer’s I test were used to determine ocular surface disorder. Use of more sensitive tests such as corneal and conjunctival staining, tear film break up time and tear osmolarity may have detected more individuals with ocular surface disorders, and may also have demonstrated structural/physiological anomalies of the ocular surface. However, as this was a community-based study, tests were chosen based on the non-requirement of sophisticated clinical instruments and investigations. Secondly, we were unable to assess the actual duration and concentration of air pollution exposure in our study participants. Measurement of the PM _2.5_ and NO _2_ concentration, along with a range of other ocular surface disorder diagnostic tests similar to that of few previous studies ^20^, would have provided us a better understanding of the association between air pollution and ocular surface disorders. Thirdly, a comparison with a control group of individuals who were not exposed to such level of air pollution or exposed to a lower level of air pollution would have allowed us to confirm that the ocular symptoms were primarily due to air pollution. Finally, we advise researchers to interpret the findings of this study with caution because of the inherent limitations of the cross-sectional nature of this study. Nevertheless, this study was the first step toward generating awareness, and exploring symptoms related to ocular surface disorder in this more exposed population. Future large-scale, longitudinal studies along with the inclusion of comprehensive tests of air pollution and ocular surface disorders are necessary to explore the detailed extent ocular surface anomalies in this population. Necessary precautions need to be taken in order to protect the ocular health of people exposed to outdoor air pollution.

## Conclusion

Traffic police officers of Kathmandu valley have a high prevalence of ocular surface complaints, which do not correlate well with the subjective tear secretion test. The duration of job appears to somewhat contribute to the increasing symptoms. In the meantime, the use of protective sunglasses ^21^ and regular eye consultations for people who are exposed to outdoor air pollution is recommended. More importantly, the government must implement new rules to reduce the levels of outdoor air pollution.

## Data availability

The data referenced by this article are under copyright with the following copyright statement: Copyright: © 2018 Paudel N et al.

Data associated with the article are available under the terms of the Creative Commons Zero "No rights reserved" data waiver (CC0 1.0 Public domain dedication). 

Dataset 1: Data on the ocular surface symptoms among individuals exposed to ambient levels of air pollution. DOI: 10.5256/f1000research.13483.d188591 ^18^

## Supplementary material

**Supplementary File 1**: OSDI questionnaire
