# Supplementary material for: Ocular surface symptoms among individuals exposed to ambient levels of traffic derived air pollution – a cross-sectional study
Source: F1000Res. 2018 Sep 18;6:2167. Originally published 2017 Dec 21. [Version 2] doi: 10.12688/f1000research.13483.2 (PMC6178909; doi:10.12688/f1000research.13483.2)
Supplement: Supplementary file 2 [file f1000research-6-17803-s0001.tgz › 3f0899e4-7895-4d1d-b267-713253df4b62_supp_file_OSDI_questionnaire.docx]

**Ocular Surface Disease Index© (OSDI©)**

Ask your patients the following 12 questions, and circle the number in the box that best represents each answer. Then, fill in boxes A, B, C, D, and E according to the instructions beside each.

| **Have you experienced any of the following *during the last week?*** | **All of the time** |  | **Most of the time** |  | **Half of the time** |  | **Some of the time** |  | **None of the time** | |
| --- | --- | --- | --- | --- | --- | --- | --- | --- | --- | --- |
| 1. Eyes that are sensitive to light? . . | 4 |  | 3 |  | 2 |  | 1 |  | 0 |  |
| 2. Eyes that feel gritty? . . . . . . . . . . . | 4 |  | 3 |  | 2 |  | 1 |  | 0 |  |
| 3. Painful or sore eyes? . . . . . . . . . . | 4 |  | 3 |  | 2 |  | 1 |  | 0 |  |
| 4. Blurred vision? . . . . . . . . . . . . . . . | 4 |  | 3 |  | 2 |  | 1 |  | 0 |  |
| 5. Poor vision? . . . . . . . . . . . . . . . . . | 4 |  | 3 |  | 2 |  | 1 |  | 0 |  |

Subtotal score for answers 1 to 5

**(A)**

| **Have problems with your eyes limited you in performing any of the following *during the last week?*** | **All of the time** |  | **Most of the time** |  | **Half of the time** |  | **Some of the time** |  | **None of the time** | | **N/A** | | |
| --- | --- | --- | --- | --- | --- | --- | --- | --- | --- | --- | --- | --- | --- |
| 6. Reading?. . . . . . . . . . . . . . . . . . . . | 4 |  | 3 |  | 2 |  | 1 |  | 0 |  |  | N/A |  |
| 7. Driving at night? . . . . . . . . . . . . . . | 4 |  | 3 |  | 2 |  | 1 |  | 0 |  |  | N/A |  |
| 8. Working with a computer or  bank machine (ATM)?. . . . . . . . . . | 4 |  | 3 |  | 2 |  | 1 |  | 0 |  |  | N/A |  |
| 9. Watching TV? . . . . . . . . . . . . . . . . | 4 |  | 3 |  | 2 |  | 1 |  | 0 |  |  | N/A |  |

Subtotal score for answers 6 to 9

**(B)**

| **Have your eyes felt uncomfortable in any of the following situations *during the last week?*** | **All of the time** |  | **Most of the time** |  | **Half of the time** |  | **Some of the time** |  | **None of the time** | | **N/A** | | |
| --- | --- | --- | --- | --- | --- | --- | --- | --- | --- | --- | --- | --- | --- |
| 10. Windy conditions?. . . . . . . . . . . . . | 4 |  | 3 |  | 2 |  | 1 |  | 0 |  |  | N/A |  |
| 11. Places or areas with low  humidity (very dry)? . . . . . . . . . . . | 4 |  | 3 |  | 2 |  | 1 |  | 0 |  |  | N/A |  |
| 12. Areas that are air conditioned?. . . | 4 |  | 3 |  | 2 |  | 1 |  | 0 |  |  | N/A |  |

Subtotal score for answers 10 to 12

**(C)**

**(D)**

Add subtotals A, B, and C to obtain D (D= sum of scores for all questions answered)

Total number of questions answered (do not include questions answered N/A)

**(E)**

Please turn over the questionnaire to calculate the patient’s final OSDI© score.

# Evaluating the OSDI© Score1

The OSDI© is assessed on a scale of 0 to 100, with higher scores representing greater disability. The index demonstrates sensitivity and specificity in distinguishing between normal subjects and patients with dry eye disease. The OSDI© is a valid and reliable instrument for measuring dry eye disease (normal, mild to moderate, and severe) and effect on vision-related function.

# Assessing Your Patient’s Dry Eye Disease


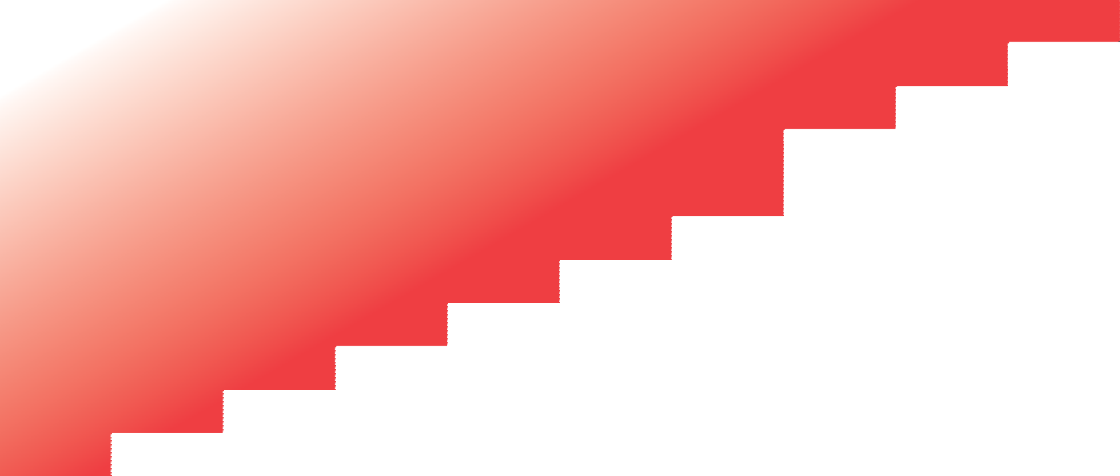
Use your answers D and E from side 1 to compare the sum of scores for all questions answered (D) and the number of questions answered (E) with the chart below.* Find where your patient’s score would fall. Match the corresponding shade of red to the key below to determine whether your patient’s score indicates normal, mild, moderate, or severe dry eye disease.

Number of Questions Answered (E from Side 1)

| **12** | **10.4** | **20.8** | **31.3** | **41.7** | **52.1** | **62.5** | **72.9** | **83.3** | **93.8** | **100.0** |
| --- | --- | --- | --- | --- | --- | --- | --- | --- | --- | --- |
| **11** | **11.4** | **22.7** | **34.1** | **45.5** | **56.8** | **68.2** | **79.5** | **90.9** | **100.0** |  |
| **10** | **12.5** | **25.0** | **37.5** | **50.0** | **62.5** | **75.0** | **87.5** | **100.0** |  |  |
| **9** | **13.9** | **27.8** | **41.7** | **55.6** | **69.4** | **83.3** | **97.2** |  |  |  |
| **8** | **15.6** | **31.3** | **46.9** | **62.5** | **78.1** | **93.8** | **100.0** |  |  |  |
| **7** | **17.9** | **35.7** | **53.6** | **71.4** | **89.3** | **100.0** |  |  |  |  |
| **6** | **20.8** | **41.7** | **62.5** | **83.3** | **100.0** |  |  |  |  |  |
| **5** | **25.0** | **50.0** | **75.0** | **100.0** |  |  |  |  |  |  |
| **4** | **31.3** | **62.5** | **93.8** |  |  |  |  |  |  |  |
| **3**  **2**  **1** | **41.7 83.3** *Values to determine dry eye severity calculated using the OSDI© formula.  **62.5** OSDI© = (sum of scores) x 25  (# of questions answered) | | | | | | | | | |
| **5** | | **10** | **15** | **20** | **25** | **30** | **35** | **40** | **45** | **48** |

Sum of Scores for All Questions Answered (D from Side 1)

Normal Mild Moderate Severe

Adapted from Allergan.
