# Supplementary material for: Ocular surface symptoms among individuals exposed to ambient levels of traffic derived air pollution – a cross-sectional study
Source: F1000Res. 2018 Sep 18;6:2167. Originally published 2017 Dec 21. [Version 2] doi: 10.12688/f1000research.13483.2 (PMC6178909; doi:10.12688/f1000research.13483.2)
Supplement: Peer review history

# Peer review: Referee response for version 2

**DOI:** 10.5256/f1000research.17803.r38477

**Published:** 2018-10-08

**Authors:** Luc LR Int Panis

**Affiliations:** 1 Environmental Risk and Health, Flemish Institute for Technological Research (VITO), Mol, Belgium; 2 Transportation Research Institute, Hasselt University, Hasselt, Belgium

I thank the authors for taking into account my suggestions and concerns to the extent possible.

I have read this submission. I believe that I have an appropriate level of expertise to confirm that it is of an acceptable scientific standard.

# Peer review: Referee response for version 2

**DOI:** 10.5256/f1000research.17803.r38478

**Published:** 2018-09-21

**Authors:** Monique Matsuda

**Affiliations:** 1 Laboratory of Ophthalmology, School of Medicine, University of São Paulo, São Paulo, Brazil

The authors reviewed the article as suggested. I have no further comments.

 I approve the article for indexing.

I have read this submission. I believe that I have an appropriate level of expertise to confirm that it is of an acceptable scientific standard.

# Peer review: Referee response for version 1

**DOI:** 10.5256/f1000research.14641.r35769

**Published:** 2018-08-20

**Authors:** Monique Matsuda

**Affiliations:** 1 Laboratory of Ophthalmology, School of Medicine, University of São Paulo, São Paulo, Brazil

The study evaluated the eye symptoms and the lacrymal production of traffic police officers in Kathmandu, Nepal, a city with high levels of air pollution.

 As the study refers to the effects of air pollution on the ocular surface of traffic police officers, it is necessary to mention the average concentration of air pollutants during August 2017, mainly PM _2.5 _and NO _2_. Vehicles are generally the main responsible for the emission of nitrogen oxides, since in Kathmandu vehicular traffic seems to be very intense, the additional information about these air pollutants is essential as part of the study.

 In addition to the air pollutants, I suggest mentioning the meteorological data, such as humidity and temperature, since these climatic factors may influence the clinical parameters of the ocular surface.

 The study was conducted during the hot and rainy season. In this period there is an increase of allergies and conjunctivitis and among the symptoms, an increased frequency of itching, foreign body sensation and photophobia. In addition, the use of fans and air conditioning is greater. All these factors could influence and favor the appearance of symptoms and the increase of OSDI score. Thus, in future studies, it would be interesting to carry out the same tests and ophthalmologic examinations during  the dry season in the same group of traffic police officers.

 Besides that, OSDI checks the symptoms during the last week. The reproducibility of symptoms of the OSDI questionnaire at different periods could be indicative of the prevalence of symptoms for a long period due to air pollution exposure and  could be more certainty correlate with the working time in the traffic. I suggest that the correlation between the OSDI score and working time should be mentioned with caution in the text, once this is a cross-sectional study.

 I recommend  the availability of an OSDI table describing the frequency of symptoms in the attachment.

 The authors mentioned very well the limitations of the study. Despite the application of only two ophthalmological parameters (OSDI questionnaire and Schirmer test), I recommend the indexing of the article since studies about the effects of air pollution in areas of high air pollutant levels, as in Kathmandu, it is necessary to evidence its effects on the ocular surface and to strengthen public policies in this area.

 I suggest minor language revision.

I have read this submission. I believe that I have an appropriate level of expertise to confirm that it is of an acceptable scientific standard, however I have significant reservations, as outlined above.

# Response

**Published:** 2018-09-11

**Authors:** Nabin Paudel

**Affiliations:** Drishti Eye Care Center, Nepal

Dear Dr Matsuda, 

 Thank you for your time to review our manuscript and providing us with an opportunity to revise the manuscript.  We have endeavoured to address each comment and suggestions. Please see below.

**Comment: *"As the study refers to the effects of air pollution on the ocular surface of traffic police officers, it is necessary to mention the average concentration of air pollutants during August 2017, mainly PM _2.5 _and NO _2_. Vehicles are generally the main responsible for the emission of nitrogen*** ***oxides,*** ***since in Kathmandu vehicular traffic seems to be very intense, the additional information about these air pollutants is essential as part of the study."***

 Response: Thank you very much for your comment. We agree with the comments. We had already provided the August 2017 data on PM _2.5_ and PM _10_ concentration in the first version. As there is no national mechanism to measure the NO _2_ system in Nepal the data from August 2017 is not available. However, in our latest version, we have included the NO _2_ data of 2015. Please refer to the introduction. 

***Comment: In addition to the air pollutants, I suggest mentioning the meteorological data, such as humidity and temperature, since these climatic factors may influence the clinical parameters of the ocular surface.***

 Response: Thank you. The meteorological data has now been added. 

***Comment:  The study was conducted during the hot and rainy season. In this period there is an increase*** ***of*** ***allergies and conjunctivitis and among the symptoms, an increased frequency of itching, foreign body sensation and photophobia. In addition, the use of fans and air conditioning is greater. All these factors could influence and favor the appearance of symptoms and the increase of*** ***OSDI*** ***score. Thus, in future studies, it would be interesting to carry out the same tests and ophthalmologic examinations*** ***during the*** ***dry season in the same group of traffic police officers.***

 Response: We absolutely agree with the comment. We have included these factors in the discussion. We also agree that it would be interesting to carry out the same tests and ophthalmologic examination during the dry season in the same group to gain a better understanding of the causative factors of ocular symptoms in this population. We are planning such a study at the moment. 

***Comment:*** ***Besides that, OSDI checks the symptoms during the last week. The reproducibility of symptoms of the OSDI questionnaire at different periods could be indicative of the prevalence of symptoms for a long period due to air pollution exposure and could be more certainty correlate with the working time in the traffic. I suggest that the correlation between the OSDI score and working time should be mentioned with caution in the text, once this is a cross-sectional study.***

 Response: Thank you very much for this close observation. We have mentioned the cross-sectional nature of the study as one of the limitations and hence have advised that caution must be applied before interpreting the research findings. 

***Comment: I*** ***recommend the*** ***availability of an OSDI table describing the frequency of symptoms in the attachment.***

 Response: We have attached the OSDI form as an attachment. 

***Comment: The authors mentioned very well the limitations of the study. Despite the application of only two ophthalmological parameters (OSDI questionnaire and Schirmer test), I recommend the indexing of the article since studies about the effects of air pollution in areas of high air pollutant levels, as in Kathmandu, it is necessary to*** ***evidence*** ***its effects on the ocular surface and to strengthen public policies in this area.***

 Response: We are thankful for your kind comments.

# Peer review: Referee response for version 1

**DOI:** 10.5256/f1000research.14641.r31452

**Published:** 2018-06-22

**Authors:** Luc LR Int Panis

**Affiliations:** 1 Environmental Risk and Health, Flemish Institute for Technological Research (VITO), Mol, Belgium; 2 Transportation Research Institute, Hasselt University, Hasselt, Belgium

This paper describes a limited analysis of eye symptoms in a small group of traffic police officers in Kathmandu, Nepal. Despite the limitations of the cross-sectional set-up and limited resources, this analysis merits publication because of the small volume of studies on ophthalmological effects of air pollution and the health effects of air pollution in non-western countries with extremely high air pollution exposures.

 The article would benefit from a brief description of concentrations of other common (gaseous or solid) air pollutants (NO2, CO, BC) during August 2017 and annual averages in the previous years in Kathmandu if available.

 The total number of questions in the OSDI questionnaire could be mentioned to facilitate the interpretation of the formula used.

 The description of the Schirmer I test is too brief to be easily understood by air pollution scientists who are no experts in ophthalmology. To consider only values <5 mm as abnormal seems to be a very strict definition e.g. compared to results presented by Karampatakis et al. ^1^. Based on RE & LE combined the % of abnormal tests is less than 15% (not 17%).

 Also there is no explanation about the OSDI of the 9% of participants that did not undergo a Schirmer I test, this could potentially lead to biased results.

 In Figure 1 the categories of Mild (23%) and Moderate (20%) seem to have been mixed up. The authors should double check whether this also happened with other categorizations (not available from the provided data) and repeat the statistical analysis if necessary. By providing more original (not categorized) data reanalysis of the data by other researchers would also be facilitated.

 The methods section does not mention that a paired t-test was used to compare results of the Schirmers I test for RE and LE.

 Because of the cross-sectional set-up of the study it would be more prudent to avoid the word ‘implies’ in the discussion and instead use ‘suggests’.

 The authors provide a good assessment of some of the limitations/weaknesses of their study. Including the lack of a control group, lack of personal/detailed exposure data or on groups with a different level of exposure and lack of more sophisticated eye tests. The inherent limitations of the cross-sectional set-up should also be mentioned.

 The study population of traffic police officers could perhaps better be characterized as a ‘more exposed’ population instead of ‘vulnerable’ unless there would be a reason why these adults would be more vulnerable than other population groups.

 With respect to their recommendation of wearing sunglasses the authors should provide a reference to a source providing evidence for the benefits of such an intervention. Were the traffic police officers questioned on the frequency of use of sunglasses during work?

 Some minor language issues could be corrected.

I have read this submission. I believe that I have an appropriate level of expertise to confirm that it is of an acceptable scientific standard, however I have significant reservations, as outlined above.

## References

[1] KarampatakisVKaramitsosASkriapaAPastiadisG: Comparison between normal values of 2- and 5-minute Schirmer test without anesthesia. *Cornea*.2010;29(5) : 10.1097/ICO.0b013e3181c2964c 497-501 10.1097/ICO.0b013e3181c2964c 20299972

# Response

**Published:** 2018-09-11

**Authors:** Nabin Paudel

**Affiliations:** Drishti Eye Care Center, Nepal

Dear Dr Panis, 

 Thank you very much for taking the time to review our manuscript. We have done our best to address your comments. We hope that our reply is satisfactory and that we have responded to, and dealt with, all comments adequately. Please see responses below:

***Comment: This paper describes a limited analysis of eye symptoms in a small group of traffic police officers in Kathmandu, Nepal. Despite the limitations of the cross-sectional set-up and limited resources, this analysis merits publication because of the small volume of studies on ophthalmological effects of air pollution and the health effects of air pollution in non-western countries with extremely high air pollution exposures.***

 Response: Thank you very much for your kind comments. We agree with the limitations of the study but hope that this study will be the first one to raise awareness among affected individuals and concerned parties regarding the effect of air pollution on ocular health. 

***Comment: The article would benefit from a brief description of concentrations of other common (gaseous or solid) air pollutants (NO2, CO, BC) during August 2017 and annual averages in the previous years in Kathmandu if available.***

 Response: Thank you very much. We apologise for the unavailability of the NO _2_, CO and BC data for the month of August 2017 as Nepal does not have a national mechanism to collect these data. We have gathered as much relevant information as we can from the published literature and have incorporated in the latest version. 

***Comment: The total number of questions in the OSDI questionnaire could be mentioned to facilitate the interpretation of the formula used.***

 Response: This has now been incorporated with the OSDI questionnaire attached as a supplementary file. 

**Comment: The description of the Schirmer I test is too brief to be easily understood by air pollution scientists who are no experts in ophthalmology. To consider only values <5 mm as abnormal seems to be a very strict definition e.g. compared to results presented by Karampatakis et al. ^1^**

 Response: Thank you for this thoughtful comment. We have included some additional information regarding the Schirmer's test. The value of <5mm for abnormal was based on its diagnostic accuracy. This has been briefly mentioned in the manuscript. 

***Comment: Based on RE & LE combined the % of abnormal tests is less than 15% (not 17%).*** ***Also*** ***there is no explanation about the OSDI of the 9% of participants that did not undergo a Schirmer I test, this could potentially lead to biased results.***

 Response: Thank you for pointing this out. We have rectified the percentage. We apologise for the lack of clarification regarding the 91% who underwent the Schirmer Test. This data was based on an earlier data analysis. We did not include the data from those patients who had incomplete information hence they were not analysed in this study. Only participants who had both the Schirmer's and OSDI scores were included in the study. 

***Comment*** ***:In*** ***Figure 1 the categories of Mild (23%) and Moderate (20%) seem to have been mixed up. The authors should double check whether this also happened with other categorizations (not available from the provided data) and repeat the statistical analysis if necessary. By providing more original (not categorized) data reanalysis of the data by other researchers would also be facilitated.***

 Response: We have now rectified the percentage. This was an error during plotting the graph. We confirm that this has not affected any other analysis. The dataset file consists of all the information that we collected and were relevant to the study project. The categorisation of age group was suggested by the editorial office. 

***Comment: The methods section does not mention that a paired t-test was used to compare*** ***results*** ***of the Schirmers I test for RE and LE.***

 Response: Thank you very much. We have mentioned it now. 

***Comments: Because of the cross-sectional set-up of the*** ***study*** ***it would be more prudent to avoid the word ‘implies’ in the discussion and instead use ‘suggests’.***

  Response: We have changed to suggests as recommended. 

***Comments: The authors provide a good assessment of some of the limitations/weaknesses of their study. Including the lack of a control group, lack of personal/detailed exposure data or on groups with a different level of exposure and lack of more sophisticated eye tests. The inherent limitations of the cross-sectional set-up should also be mentioned.***

 Response: Thank you so much for your kind comments. We have added the cross-sectional nature of the study as one of the limitations and have advised readers that the results must be interpreted with caution. 

***Comments: The study population of traffic police officers could perhaps better be characterized as a ‘more exposed’ population instead of ‘vulnerable’ unless there would be a reason why these adults would be more vulnerable than other population groups.***

 Response: We have changed vulnerable to more exposed. Thank you. 

***Comment: With respect to their recommendation of wearing*** ***sunglasses*** ***the authors should provide a reference to a source providing evidence for the benefits of such an intervention. Were the traffic police officers questioned on the frequency of use of sunglasses during work?***

 Response: We have added a reference that reports the beneficial effect of glasses on ocular surface disorders such as dry eyes. Unfortunately, we did not ask any questions regarding the frequency of the use of sunglasses during work but will definitely consider for future studies. 

******

***Comments: Some minor language issues could be corrected.***

 Response: We have attempted to reduce such issues as much as possible.
